# Key competencies of students with autism spectrum disorders: Perspectives of Chinese teachers and parents

**DOI:** 10.3389/fpsyg.2022.1054249

**Published:** 2022-12-22

**Authors:** Shuqin Cao, Yixin Wang, Xiaofang Yang, Qiqin Jin, Ying Hui-Michael, Dongjie Xie

**Affiliations:** ^1^School of Special Education, Zhejiang Normal University, Hangzhou, China; ^2^Changhe Kindergarten, Hangzhou, China; ^3^Department of Special Education, Rhode Island College, Providence, RI, United States

**Keywords:** autism spectrum disorders, key competencies, learning outcomes, teachers, parents

## Abstract

Well-defined key competencies for students with autism spectrum disorders (ASD) help develop curriculum and pedagogies that emphasize what students with ASD are expected to learn, to know and to do. Most of the current research on the key competencies of ASD is theoretical and based on the social and cultural backgrounds of western countries. The key competencies defined by most of the research lack of the support of empirical evidence. This study sought to identify the key competencies of school-age students with ASD from the perspectives of teachers and parents. Based on the review of existing key competencies frameworks, a key competencies instrument that consisted of 76 learning outcome items in eight domain areas was developed. An online survey to explore the teachers’ and parents’ views of the key competencies was conducted with 1,618 teachers and 2,430 parents of students with ASD across China. The results showed that teachers believed that the key competencies should consist of eight domain areas including social-communication, learning skills, healthy living, play, motor, emotion, sensory processing, and cognition, while the cognition related competencies were not recognized by parents. The competencies in social-communication, learning skills, healthy living had higher variance contribution. From the perspective of teachers, the variance contribution of social communication was the highest, while from the perspective of parents, the variance contribution of learning skills was the largest. Taken together, the key competencies framework for students with ASD should include eight dimensions and 75 learning outcome items. The similarities and differences between the perspectives of the two group were discussed. The findings could provide empirical data to assist in developing educational guidelines and guide the development of models of support for students with ASD.

## 1. Introduction

With the increasing prevalence of autism, education for students with autism spectrum disorders (ASD) has become a major focus of the education policy and research worldwide. The Autism CARES Act of 2019 enacted by the Department of Health & Human Services in the United States (U.S. Department of Health & Human Services Interagency Autism Coordinating Committee, 2019) incorporates autism education into the law. The National Strategy for Autistic Children, Young People and Adults: 2021 to 2026 promulgated by the British government elevates autism education to a national strategic position (Department of Health and Social Care and Department for Education, 2021). The 14th Five-Year Plan of Action for the Development and Enhancement of Special Education issued by the Chinese Ministry of Education (2022) also specifically puts forward requirements for autism education. These polices indicate the consensus that students with ASD should have access to high quality education.

To ensure high quality education, many countries and international organizations including the U.S., the U.K., Singapore, and the Organization for Economic Co-operation and Development (OECD), have proposed preparing students to develop key competencies as an important educational goal in the 21st century (Liu, 2017). Key competencies represent a set of desired learning outcomes integrating the knowledge, skills, attitudes and values, which are necessary for personal fulfillment, development throughout life, social inclusion and employment (Rychen and Salganik, 2007). While there are well-documented key competencies (e.g., learning skills, critical thinking, communication, creativity and collaboration) to prepare students for the 21st century, the key competencies for students with special education needs, such as the students with ASD have been ignored at a large extent. And it is urgent to clarify the key competencies for students with ASD to provide high quality education.

Some existing research shared what competencies students with ASD should learn. The Autism Education Trust in the U.K. developed a progression framework for students with ASD [Autism Education Trust (AET), 2019]. The framework comprises eight main learning areas, including social understanding and relationships, learning and engagement, communication and interaction, emotional understanding and self-awareness, sensory processing, healthy living, interests, routines and processing, and independence and community participation. Each of the eight learning areas is structed with multiple sub-learning areas, learning outcomes and example of learning outcomes. For example, under the area of communication and interaction, it has a sub-learning area as engaging in interaction. A learning outcome in interaction is to “share attention with adult”; and an example of the learning outcome is to “accept adult sharing an activity.” Therefore, the framework was suggested to provide the support to guide practitioners to develop learning outcomes, implement intervention and monitor learning progress.

Using content analysis, Cao et al. (2019) analyzed and compared 10 clinical guidelines for ASD in developed countries and regions, for example, New Zealand Autism Spectrum Disorder Guideline. They found that the learning goals for the students with ASD were mainly distributed in three major competency domains: self-improvement, social skills, and tool use. Specifically, the social skills domain focused on skills in social language, socialization, and challenging behaviors. The self-improvement domain focused more on learning goals related to attention, cognition, motor development, and sensory response, followed by physical development, self-management, and imitation. There were fewer goals in the tool use domain such as academic skills. Some guidelines focused on the remediation to improve their competencies in social communication and stereotypic behaviors. And others focused on holistic education that improved students’ overall growth and develop the potential. Most of the guidelines shared competencies domain areas without clarifying the specific outcomes.

Hume et al. (2021) systematically reviewed 972 evidence-based practices (EBPs) studies on children and youth with ASD from 1990 to 2017. It was found that 13 competency areas (i.e., academic/pre-academic performance, adaptive/self-help, challenging/interfering behavior, cognition, communication, joint attention, mental health, motor, play, self-determination, school readiness, social and vocational skills) were addressed in these EBPs studies. While the communication, social, and challenging/interfering behavior related areas were studied most often, there was increasing attention given to academic skills, vocational skills, and mental health.

However, these findings only revealed the expectations on students with ASD in the western cultural context, while the situation may be different in Chinese cultural context. For example, in one transnational online training program for parents of children with ASD in China, when American trainers thought that some repetitive behaviors were normalized, the parents had opposing ideas and hoped their children look like other kids and “fit in society” (McDevitt, 2021). In addition, there is a culture of “collectivism” in Chinese education system. Although the individuality of students is acknowledged, students are firstly as one part of the collective group and must obey the required classroom principles, orders and conditions (Zhu and Li, 2020).

The only documented framework of key competencies for Chinese students with ASD was conducted by Jin et al. (2022). Jin and her colleagues were commissioned by the Ministry of Education of China in 2016 to develop the framework of key competencies and values for Chinese school-age students with ASD. Taking Chinese social-cultural backgrounds and educational system into consideration, and based on the analysis of international guidelines for ASD (e.g., North Dakota Guidelines for Serving Students with Autism Spectrum Disorder in Educational Settings), the framework covered eight key competencies areas, including health, personal independence, earning skills, cognition, interpersonal relationships, communication, play, and community participation. In addition, the framework offers numerous learning outcomes to clarify what to teach and assess. The learning outcomes were created as measurable statements by using action verbs that articulated what the knowledge and skills that student should learn. For example, “Choose and participate in leisure activities such as shopping, recreation, sports, and vacation” in health. But it still lacks empirical evidence with incorporating perspectives from the primary educators of students with ASD.

Teachers and parents are the key stakeholders in the education, and their attitudes, choices and needs are crucial to frame the key competencies for students with ASD. But the main educational place for parents is in the family environment, and the content is mainly focused on living. The teachers’ educational place is mainly in the collective school environment, which needs to face the students’ academic and interpersonal communication situations. Different identities and educational settings may lead to different educational expectations for the development of students with ASD. Research has also shown that parents and professionals have different perspectives on the education for ASD (Nissenbaum et al., 2002; Gabovitch and Curtin, 2009). Although parents and teachers share common concerns in many areas, teachers tend to focus more on the restricted, repetitive, and stereotyped behaviors of students with ASD, while parents place more importance on academics (Azad and Mandell, 2016). Similarly, Dillenburger et al. (2010) found that professionals were concerned about externalizing behaviors, while parents were more worried about behaviors commonly associated with ASD, such as deficits in interaction, play, social skills and communication. The value judgment and educational behaviors of teachers and parents on the development of students with ASD are the basis for their perceptions of student development (Yao, 2014). Since the significance of this study is to shape the key competencies framework for students with ASD, it is essential to clarify teachers’ and parents’ expectations.

Therefore, this study aims to identify the perspectives of Chinese teachers and parents regarding key competencies for Chinese school-age students with ASD. Two research questions were addressed in this study:

*Q1*: What are the key competencies identified by Chinese parents and teachers of students with ASD?

*Q2*: What are the similarities and differences between the parents and teachers regarding the identified key competencies?

## 2. Materials and methods

### 2.1. Participants

Using a stratified random sampling method, teachers and parents who met the participant criteria were recruited from different regions (i.e., Eastern, Central, Western, and Northeast) to participate in the study. The participant criteria included: (1) teachers or parents of students diagnosed with ASD by clinical experts according to Autism Diagnostic Observation Schedule (ADOS), 11th revision of the International Classification of Diseases (ICD-11) or Diagnostic and Statistical Manual of Mental Disorders, 5th edition (DSM5), and (2) students were between 6 and 18 years old. The samples were stratified proportionately based on the number of special schools in each region. The online survey was tested including, clarifying introduction, checking the format, and reviewing questionnaires. The online survey was first distributed to the special education guidance centers or relevant administrative departments in all regions including 30 provinces, autonomous regions, and municipalities. The centers and departments then administered the online survey to potential participants. 4,446 responses were return with the valid as 91.05%. The 4,048 valid responses included 1,618 from teachers and 2,430 from parents. All participants provided informed consent before taking part in the study. The demographic information is shown in Tables 1, 2.

**Table 1:** Teacher demographics (*n* = 1,618).

Variables		Frequency	%
School type	Special education school	1,438	88.88%
	General school	180	11.12%
Region	Eastern	706	43.63%
	Central	487	30.10%
	Western	323	19.96%
	Northeast	102	6.30%
Gender	Male	299	18.48%
	Female	1,319	81.52%
Grade	Grade 1–3	750	46.35%
	Grade 4–6	472	29.17%
	Grade 7–9	344	21.26%
	High school	52	3.21%
Age	20–25 years	249	15.39%
	26–30 years	324	20.02%
	31–35 years	311	19.22%
	36–40 years	227	14.03%
	41–45 years	209	12.92%
	45–50 years	177	10.94%
	Above 50 years	121	7.48%
Years of teaching	1–5 years	535	33.07%
	6–10 years	311	19.22%
	11–15 years	168	10.38%
	16–20 years	153	9.46%
	21–25 years	185	11.43%
	26–30 years	145	8.96%
	Above 30 years	121	7.48%

**Table 2:** Parent demographics (*n* = 2,430).

Variables		Frequency	%
Region	Eastern	1,029	42.35%
	Central	589	24.24%
	Western	676	27.82%
	Northeast	136	5.60%
Education level	Elementary school and below	227	9.34%
	Junior high school	974	40.08%
	High school or secondary school	581	23.91%
	University	606	24.94%
	Master and above	42	1.73%
	Others	310	12.76%
School placement of child	Special education school	1,461	60.12%
	General school	969	39.88%
Gender of child	Male	1,631	67.12%
	Female	799	32.88%
Age of child	6–9 years	951	39.14%
	10–12 years	873	35.93%
	13–15 years	408	16.79%
	16–18 years	198	8.15%
Grade of child	Grade 1–3	1,238	50.95%
	Grade 4–6	889	36.58%
	Grade 7–9	271	11.15%
	High school	32	1.32%
IQ level of child	IQ > 90	350	14.40%
	IQ > 70	695	28.60%
	IQ:70–55	298	12.26%
	IQ:55–40	587	24.16%
	IQ:40–25	421	17.33%
	IQ < 25	79	3.25%

### 2.2. Instrument

The self-completion survey consisted of eight competency domains and 76 learning outcome items (see Supplementary material). The eight domains and 76 learning outcome items were generated based on careful reviews of several competencies frameworks [e.g., Autism Education Trust (AET), 2019]. The Key Competencies and Values constructed by Jin et al. (2022) was the most suitable resource for the survey development. The learning outcomes in the framework were not only concrete, but also provided socially relevant competencies. For example, “Get along with others in familiar or public settings.”

Two round of focus groups was used to shape the content and form of the survey. Eight professionals (1 associate professor, 7 teachers of students with ASD, and 8 graduate students) in the field of special education were consulted for the first round of evaluation. Fifteen professionals (12 researchers, 3 special education teachers, and 15 graduate students) in the field of special education were sought for the second round of evaluation.

Each of the eight domains (i.e., healthy living, personal independence, learning skills, cognition, interpersonal relationships, communication, play, and community participation) had multiple learning outcome items ranged from 5 to 15. For example, the healthy living domain presented in the first section of the survey had 15 items, and the community participation domain in the last section of the survey had 5 items. Using a five-point scale, teachers and parents rated the importance of each learning outcome for students with ASD from 1 (not important at all) to 5 (extremely important).

### 2.3. Statistical analysis

Using SPSS 23, item analysis, exploratory factor analysis, and internal consistency reliability analysis were performed on the data of teachers (*n* = 1,618) and parents (*n* = 2,430), respectively. Item analysis was first based on independent samples t-tests between the high and low groups (top 27% and bottom 27% of the total score for all items), followed by the correlation between items and the total score. Exploratory factor analysis (EFA) was conducted to explore the underlying structure of key competencies of students with ASD. In this study, principal component analysis and promax oblimin rotation method were used to explore teachers’ and parents’ understanding of the key competencies for students with ASD, respectively. We excluded the items with factor loading less than 0.45 or with high loadings of several common factors at the same time (cross loading between factors >0.2). Internal consistency reliability was measured using the Cronbach’s alpha coefficient.

## 3. Results

### 3.1. Item analysis

The independent samples t-test results showed that the difference between the high and low groups of each item was statistically significant (all *p* values <0.001, see Supplementary material), indicating that the items were well differentiated. Each item scores of the teacher and parent responses were significantly correlated with the total score (see Supplementary material). The correlation coefficients were all above 0.4.

### 3.2. Construct validity

#### 3.2.1. The perspectives of teachers

EFA was performed on 76 learning outcome items. The Kaiser-Meyer-Olkin (KMO) value was 0.987, the Bartlett’s sphericity test value was 137162.630, df = 2,850, *p* < 0. 001, indicating a structure of data suitable for factor analysis. A total of nine factors of competency domains with eigenroots >1 were obtained, and the cumulative variance contribution rate was 75.178%. Thirteen items needed to be excluded according to the exclusion principle. The second factor analysis was conducted on the remaining 63 items. The KMO value was 0.985, the Bartlett’s sphericity test value was 11066.038, df = 1953, *p* < 0.001, indicating that the factor analysis was suitable. The analysis yielded eight factors of competency domains with eigenroots >1, and the cumulative variance accounts for 75.108%. The eight factors named according to their contents were social-communication, learning skills, healthy living, play, motor, emotion, sensory processing, and cognition (see Table 3). The three factors with the highest variance contribution were social-communication (54.057%), learning skills (6.332%) and healthy living (3.893%). The correlation coefficients between the scores of each factor and the total score across all items ranged from 0.600 to 0.941, and the correlation coefficients between the factor scores ranged from 0.343 to 0.835, all *p* values <0.001.

**Table 3:** The comparison of competency domains between teachers and parents.

Factors	Teachers		Parents	
	% Variance explained	Items	% Variance explained	Items
Social-communication	54.057	59–76	6.862	59–73
Learning skills	6.332	42–49	54.523	37–49
Healthy living	3.893	18–26	4.641	15–25, 27–29
Play	2.849	50–57	2.617	50–58
Cognition	2.156	30–36		
Motor	2.101	5–8	2.547	5–7
Emotion	2.055	10–14	1.934	10–14
Sensory processing	1.665	1–4	1.650	1–3

#### 3.2.2. The perspectives of parents

EFA was also performed on 76 learning outcome items. The KMO value was 0.989, the Bartlett’s sphericity test value was 210464.156, df = 2,850, *p* < 0. 001, supporting factorability of the items in the surveys. A total of seven factors with eigenroots >1 were obtained, and the cumulative variance contribution rate was 73.227%, of which 14 items needed to be excluded. The second factor analysis was conducted on the remaining 62 items. The KMO coefficient (0.986) and Bartlett’s Test of Sphericity [χ^2^ [1891] = 167954.267, *p* < 0.001] indicated that exploratory factor analysis could be applied to the data. The analysis yielded seven factors of competency domains with eigenroots >1, and the cumulative variance contribution rate was 74.775%. The seven factors were named according to their contents (see Table 3). Among them, leaning skills (variance contribution of 54.523%), social-communication (variance contribution of 6.862%) and healthy living (variance contribution of 4.641%) had the highest explanatory power. The correlation coefficients between the average score of each factor and the total score across all items ranged from 0.504 to 0.940, and the correlation coefficients between the factors scores ranged from 0.279 to 0.940, all *p* values <0.001.

Summarizing the factors and item indicators of the above two structures, we found that the key competencies of students with ASD should cover 75 learning outcomes items and eight factors of competency domains, namely social-communication, learning skills, healthy living, play, motor, emotion, sensory processing, cognition. Although cognition was not viewed significantly important by parents, other seven domains were recognized by both teachers and parents. Fifty of the initial 76 item indicators appeared in the final structure of both teachers and parents, and were equally distributed in each factor. The primary variations being in factors such as healthy living, learning skills, and social-communication.

### 3.3. Internal consistency reliability

The Cronbach’s alpha coefficient of the 63 items identified by teachers was 0.986, and the Cronbach’s alpha coefficients of each factor of key competencies domains were greater than 0.855. The Cronbach’s alpha coefficient of the 62 items identified by parents was 0.986, and the Cronbach’s alpha coefficients of each factor were greater than 0.805.

## 4. Discussion

This study examined and constructed an inventory of key competencies for Chinese students with ASD from the perspectives of teachers and parents, respectively. The item analysis and internal consistency analysis results met the quality of psychometrics. The EFA results found that teachers and parents share common understanding about the key competencies of students with ASD, but there are also certain differences.

### 4.1. Consensus on the key competencies of students with ASD

Both teachers and parents believed that the key competencies of students with ASD should consist of social-communication, learning skills, healthy living, play, motor, emotion, and sensory processing, with the first three factors having higher explanatory power. Fifty of the initial 76 item indicators appeared in the final structure of both teachers and parents, and were equally distributed in each factor, indicating a high degree of consistency between the items and their corresponding factors.

First, both parents and teachers identified social-communication as one of the key competencies to be developed for students with ASD, with a strong emphasis on compensating for social deficits. And the social-communication factor was clustered with the ability to communicate, socialize, and be in groups. Social-communication is the most important area in which parents focus their attention (Lin et al., 2007), and parents believe that having good social relationships is the most important priority for their children’s adult lives. In the same way, teachers give social area the same attention and importance (Azad and Mandell, 2016). Kurth and Mastergeorge (2009) specifically analyzed goals in Individual Education Plan (IEP) text of students with ASD, and found that most IEP goals were for core symptoms such as communication and social interaction, see also Wilczynski et al. (2007).

Second, parents and teachers have certain expectations and requirements for the learning skills of students with ASD. Education guidelines for ASD from North Dakota, Virginia, Kansas, the United Kingdom, and Taiwan, China also consider the academic competencies as important goals (Cao et al., 2022). In addition, some studies also suggest that acquiring key academic skills, such as the abilities to read and write, are important good outcomes for some adults with ASD (Wittemeyer et al., 2011).

Further, the key competencies for survival are also highly valued by parents and teachers. Healthy living relates to being able to live independently (e.g., engage in appropriate leisure activities at home, manage household goods and finances, choose and participate in leisure activities, and manage health-care). Several researches also suggested developing the living abilities of students with ASD first (Bilgin and Kucuk, 2010; Liu and Breslin, 2013; Sosnowy et al., 2018), for healthy living is essential for independent living, further education, and participation in employment in adulthood.

In addition, teachers and parents have paid similar attention to other related deficit areas, considering that the key competencies of students with ASD should also cover motor, emotion, sensory processing and play.

In this study, the motor factor involved gross, fine, balance and coordination, and physical resilience. Research suggests that children with ASD have difficulties with delayed motor skills and movement compared to typically developing children (Liu and Breslin, 2013), which needs attention.

The emotion factor involves emotional understanding, expression, regulation, regulatory control, and problem behavior management. For students with ASD, the presence of emotional problem behaviors is not only detrimental to their own development, but also interferes with teachers’ teaching and peers’ learning, and should be a priority area of improvement.

The sensory processing factor involves the competencies to respond to, tolerate, express, and manage sensations. The DSM5 includes abnormalities in sensory abilities as one of the diagnostic indicators, and six of the 10 international autism guidelines available in the United Kingdom and the United States involve sensory process (Cao et al., 2022).

The play factor involves imitation, individual play (e.g., explore toys or materials, play combination games, play cause and effect games, and play functional games), and social play (play parallel games, play joint games, etc.). The ability to play can be used as a grip for the enhancement of students with ASD. In the process of play, children have the natural and yet powerful opportunity to integrate the capacities of regulation, symbolic capacity, emotional development, and readiness for learning. In children with ASD, learning through play is seen to be more powerful than learning through adult-directed activities (Gerber, 2017).

Overall, the seven competencies areas shared by teachers and parents in this study not only responded to core deficits such as social interaction among students with ASD, but also focused on related deficit areas such as healthy living and sensory processing. Research has also shown that ASD-related stakeholder groups believe that social-communication, learning skills, healthy living are important for a better life with ASD in adulthood (Wittemeyer et al., 2011).

### 4.2. Complementary perspectives between teachers and parents

According to the results, teachers and parents still have different views on the key competencies that students with ASD should possess. Overall, the results of teachers’ data showed that social-communication had the highest variance contribution followed by learning skills and healthy living, while parents’ data analysis suggested that learning skills had the largest explanatory power followed by social-communication and healthy living. In addition, teachers pay more attention to cognition.

This discrepancy reflects the different perceptions and expectations of the two groups for students with ASD. In schools, teachers are more likely to identify students’ social deficits and thus place more emphasis on social skills. Whereas a parent may be interacting with a child, the parent is more forgiving of the child’s social issues and thus more likely to help the child improve his or her learning skills. It has also been shown that parents and teachers do not always have the same views on ASD (Barnhill et al., 2000; Azad and Mandell, 2016).

Chinese parents of children with ASD were concerned about learning quality such as motivation, self-regulation and reflection. They have higher expectations on their children’s academic related competencies. Chinese culture places a particular emphasis on academic achievement (Chao, 1994), and children’s academic attainment is considered a key factor reflecting good future or respectability (Leung and Shek, 2011). And the one-child policy has led to high expectations for individual children’s academic and career achievements (Zhang et al., 2015). In addition, some Chinese parents may view their children’s learning difficulties as a reflection of inadequate effort and self-discipline or unsuccessful training attempts (Tews and Merali, 2008).

However, the teacher’s work conditions “filtered” the effect of cultural traditions. The teacher’s work situations can influence or even limit teacher’s instructional choices (Cuban, 1987). In China, school-age children with ASD are mostly placed in special education schools and regular classrooms in general schools for children with high-functioning (Hu and Fan, 2016). The majority of teachers in this study (88.88%) worked in special education schools and had to interact with children who had both ASD and intellectual disabilities, which may have resulted in lower learning quality expectations. In addition, these teachers all worked in a typically age-graded classroom structure. It implies that one teacher has to deal with 12 students with special education needs (including intellectual disability and ASD) simultaneously, and the teacher also need to complete the corresponding teaching tasks within the limited time. Due to ASD characteristics, students with ASD are prone to challenging behaviors in group classrooms, which can interrupt the class. Therefore, it is more crucial for teachers to ensure an orderly operation of the classroom rather than developing students’ higher-order learning qualities.

This may also explain why teachers emphasize the cognition competencies of students with ASD, while parents do not focus on them. The cognition factor includes cognitive flexibility (e.g., flexibly switch attention between people, objects, situations, and activities; transfer between sessions; adapt to new changes in the environment) and thinking skills (e.g., solve problems). These competencies are extremely important for participation in the group classroom. But parents may not pay attention to these because they may be accustomed to students’ stereotyped interests and behaviors at home that are disruptive to the classroom (Azad and Mandell, 2016). Moreover, cognition may be relatively abstract to parents.

In terms of healthy living, parents focused on daily life related skills such as self-care (items 15–17) and adolescent problem handling (items 27–29) for students with ASD, while teachers paid less attention to those aspects. This is consistent with previous studies. Parents acknowledged that achievement of independence and happiness depends on the completion of a certain level of education and the acquisition of numerous other skills such as self-help skills (Starr and Foy, 2012). Additionally, adolescents with ASD are greatly at a disadvantage due to their lack of sexual knowledge, which also poses extra social and health issues (Lehan Mackin et al., 2016). For parents of autistic teenagers, the subject of sexuality can be especially troublesome since they believe it to be an additional burden for their child (Travers and Tincani, 2010).

Interestingly, in terms of social-communication, teachers believe that students with ASD should have social responsibility, social belonging and career readiness skills, while parents believe that these areas are less important as relative items were deleted in parents’ structure. It is clear that the two groups have different values regarding the development and education of students with ASD. Teachers expect students from a collective perspective, emphasizing their participation and role in the classroom, school, and even society, and attaching importance to the socialization of students with ASD. However, parents make more demands from the standpoint of individuality, placing greater emphasis on their children’s adaptation and quality of life. This is also in line with the Chinese cultural context of “collective individualism,” which values collective responsibility in the classroom environment while also focusing on individual development.

Differences do exist between teacher and parent groups, but an important prerequisite for effective home-school partnership is that parents and teachers share their concerns and agree on the priority areas that need to be addressed (Jivanjee et al., 2007; Esquivel et al., 2008). Good collaboration is not about eliminating differences, but about understanding the needs of each group to find coexistence and synergy. Since one of the most distinctive features of ASD is heterogeneity, the framework proposed in this study is not a closed, fixed system of key competencies, but rather a maximized menu of key competencies. As a result, different perspectives must be included to make it more complete and comprehensive. Teachers and parents have different educational contexts and perspectives, but bringing them together and complementing one another result in a more cohesive, functional, and comprehensive understanding of key competencies. Combining the two perspectives, it can be concluded that the key competencies for students with ASD include social-communication, learning skills, healthy living, play, motor, emotion, sensory processing and cognition, a total of eight dimensions. This verifies the theoretically constructed framework of key competencies for students with ASD by Jin et al. (2022) and provides a specific indicator system for reference. It generally covers the basic demands of educational practice for the current development of students with ASD and the emphasis of parents and teachers, indicating that the key competencies for students with ASD are holistic in nature. It also shows that education for ASD should balance the defect remediation and potential development, balance basic survival and long-term well-being, and balance personal development and social integration.

### 4.3. The practical meaning of the key competencies framework

To date, there are few studies systematically exploring a reasonable framework of learning outcomes for students with ASD from an empirical perspective. This study adopts a “convergence” approach to develop a consensus based on the contrasting perceptions of teachers and parents in the Chinese cultural context. Results of the study will certainly have multiple implications to the field of practice.

Firstly, the key competencies framework provides a broader perspective of educational attainment for student with ASD. The framework with competency items can be used to identify appropriate educational goals and then adapt the curriculum and instruction accordingly, which will improve the learning outcomes of students with ASD in China. The framework can also enable holistic education for students with ASD by facilitating social and cultural perspectives on disability.

Secondly, the key competencies framework can help educators design and implement more targeted and effective IEPs for students with ASD, especially for those students in inclusive settings. Obviously, Chinese parents and educators have called for effective inclusive education for student with ASD. However, students with ASD in inclusive settings struggle immensely due to a lack of special education support. And Chinese educators are seeking more ready-to-use resources for educating students with ASD. The eight competencies dimensions and 75 learning outcome items provide specific samples that can be adopted for IEP goals. For example, there are 18 items under social-communication area, then teachers can locate social-communication area and choose a learning outcome item (e.g., express needs, options, events, ideas and comments) for IEP goal development.

Finally, the interrelationship of Chinese teachers’ and parents’ perspectives regarding the priority needs of students with ASD will enhance the collaboration between these two groups. The deep collaboration between educators and parents has been increasingly emphasized worldwide. For example, guidelines from the AET, North Dakota, Kansas, Washington State, and New Zealand all emphasize the necessity of parent-teacher collaboration. However, collaboration is built on shared values. This study shows that parents and teachers have their own “lens” and hold differentiated expectations for students with ASD. The key competencies framework incorporates both parents’ and teachers’ concerns, thus providing a common tool for dialogue between parents and teachers. It can facilitate shared values and shared decision-making. Since parents and teachers may have different understandings on specific indicators of the framework, training for parents and teachers may be needed to avoid mutual misunderstandings.

### 4.4. Research limitations

This study does have certain limitations. Firstly, the authenticity of the questionnaire may be compromised by filling it out online, and the lack of relevant qualitative interview data will not allow us to see deeper perceptions. Secondly, the insufficient number of teachers in the regular classroom of general schools may affect the selection of key competencies indicators. Thirdly, since teachers and parents only have diagnosis certificates of students with ASD, and do not determine the specific diagnostic criteria, this study did not distinguish the impact of different diagnostic bases.

In addition, the currently proposed key competencies framework only considers the horizontal content areas and describes only a single form of competencies, making it difficult to measure and evaluate competencies development. In order to enhance the precision, operability, and implementability of the framework, it may be necessary to draw on the relevant results of the taxonomy of educational goals to further improve the framework to clarify what students with ASD should learn, understand, and how to do.

### 4.5. Conclusion

Based on a survey of teachers and parents in China, this study systematically quantified the key competencies structure of students with ASD. A key competencies framework with 75 learning outcome items in eight domains was derived through factor analysis. Corresponding competencies items should be constructed around the areas of social-communication, learning skills, healthy living, play, motor, emotion, sensory processing, and cognition. It provides a starting point for future research on teaching curriculum and assessment based on this framework, as well as a reference for curriculum policy and educational practice.

## Data availability statement

The raw data supporting the conclusions of this article will be made available by the authors, without undue reservation.

## Ethics statement

The studies involving human participants were reviewed and approved by Human Experiment Ethics Committee of Zhejiang Normal University. The patients/participants provided their written informed consent to participate in this study.

## Author contributions

SC, YW, DX, QJ, and YH-M designed the research and wrote the manuscript. SC and XY collected and analyzed the data. All authors contributed to the article and approved the submitted version.

## Funding

This study was supported by a grant from the National Natural Science Foundation of China (32000751), the MOE (Ministry of Education in China) Project of Humanities and Social Sciences (18YJCZH199), the Natural Science Foundation of Zhejiang Province (LQ19C090003), and the National Social Science Foundation of China (BHA190143).

## Conflict of interest

The authors declare that the research was conducted in the absence of any commercial or financial relationships that could be construed as a potential conflict of interest.

## Publisher’s note

All claims expressed in this article are solely those of the authors and do not necessarily represent those of their affiliated organizations, or those of the publisher, the editors and the reviewers. Any product that may be evaluated in this article, or claim that may be made by its manufacturer, is not guaranteed or endorsed by the publisher.
