# Supplementary material for: Key competencies of students with autism spectrum disorders: Perspectives of Chinese teachers and parents
Source: Front Psychol. 2022 Dec 22;13:1054249. doi: 10.3389/fpsyg.2022.1054249 (PMC9813226; doi:10.3389/fpsyg.2022.1054249)
Supplement: Supplementary file 1 [file Table_1.DOCX]

Supplementary Material

# Table 1 *Descriptive statistics for 76 key competency items*

| Items | | Parents | | Teachers | |
| --- | --- | --- | --- | --- | --- |
|  |  | M | SD | M | SD |
| 1 | Respond appropriately to external input of sensory stimuli | 3.80 | 0.97 | 3.98 | 0.90 |
| 2 | Appropriate tolerance to sensory stimuli | 3.66 | 0.91 | 3.92 | 0.88 |
| 3 | Express sensory preferences or other sensory needs | 3.73 | 0.95 | 3.88 | 0.91 |
| 4 | Use appropriate methods to manage sensory discomfort | 3.77 | 0.99 | 3.90 | 0.95 |
| 5 | Perform basic gross motor movements | 4.08 | 0.99 | 4.19 | 0.86 |
| 6 | Perform basic hand manipulation movements | 4.14 | 0.98 | 4.25 | 0.84 |
| 7 | Maintain balance and coordination of the body | 4.07 | 0.98 | 4.16 | 0.86 |
| 8 | Demonstrate a certain level of physical ability and toughness | 3.95 | 0.95 | 3.99 | 0.86 |
| 9 | Perform motor activities in context | 3.82 | 0.95 | 3.93 | 0.85 |
| 10 | Show intent | 3.81 | 0.98 | 3.93 | 0.89 |
| 11 | Recognize and understand emotions | 3.97 | 0.96 | 4.07 | 0.89 |
| 12 | Express and respond appropriately to the emotions of others with words, expressions, and body movements | 3.98 | 0.98 | 4.12 | 0.89 |
| 13 | Regulate and control emotions | 3.93 | 0.97 | 4.11 | 0.88 |
| 14 | Manage their own challenging behaviors | 3.81 | 1.02 | 3.93 | 0.96 |
| 15 | Manage diet | 4.10 | 0.96 | 4.31 | 0.86 |
| 16 | Take care of laundry | 3.99 | 1.00 | 4.23 | 0.86 |
| 17 | Maintain grooming and hygiene | 4.19 | 0.92 | 4.34 | 0.84 |
| 18 | Manage health-care | 3.90 | 1.08 | 4.00 | 0.96 |
| 19 | Keep the home environment clean and tidy | 3.85 | 1.01 | 3.95 | 0.90 |
| 20 | Manage household goods and finances | 3.75 | 1.14 | 3.81 | 0.97 |
| 21 | Handle and maintain home safety | 4.07 | 1.07 | 4.17 | 0.94 |
| 22 | Engage in appropriate leisure activities at home | 3.89 | 0.93 | 3.96 | 0.86 |
| 23 | Choose and participate in leisure activities such as shopping, recreation, sports, and vacation | 3.77 | 0.95 | 3.82 | 0.86 |
| 24 | Use public places, facilities and services in the community | 3.78 | 0.95 | 3.85 | 0.86 |
| 25 | Knowledge and awareness of safety in community activities | 3.87 | 1.08 | 3.96 | 0.93 |
| 26 | Make self-decisions | 3.71 | 1.12 | 3.75 | 0.96 |
| 27 | Be interpersonally aware | 3.87 | 1.03 | 3.96 | 0.90 |
| 28 | Maintain appropriate interpersonal distance during adolescence | 3.94 | 1.08 | 4.12 | 0.91 |
| 29 | Cope with and manage the changes and challenges of adolescence | 3.91 | 1.13 | 4.12 | 0.94 |
| 30 | Actively develop thinking skills | 3.80 | 1.01 | 3.86 | 0.90 |
| 31 | Solve problems | 3.82 | 1.04 | 3.87 | 0.90 |
| 32 | Apply the knowledge and skills learned in generalization | 3.75 | 1.06 | 3.91 | 0.92 |
| 33 | Flexibly switch attention between people, objects, situations and activities | 3.80 | 1.03 | 3.92 | 0.91 |
| 34 | Adapt to new changes in the environment | 3.88 | 0.93 | 4.01 | 0.86 |
| 35 | Transfer between sessions | 3.82 | 0.96 | 3.92 | 0.88 |
| 36 | Participate in activities other than those of special interest | 3.75 | 0.94 | 3.84 | 0.88 |
| 37 | Stimulate and maintain motivation for learning | 3.82 | 1.01 | 3.82 | 0.90 |
| 38 | Maintain concentration during the learning process | 3.90 | 1.02 | 3.97 | 0.88 |
| 39 | Persist in completing tasks in the course of learning activities | 3.91 | 1.00 | 3.93 | 0.88 |
| 40 | Participate in negotiating learning tasks | 3.78 | 1.08 | 3.79 | 0.93 |
| 41 | Organize own learning activities | 3.73 | 1.11 | 3.78 | 0.91 |
| 42 | Reflect on and evaluate own learning activities | 3.67 | 1.14 | 3.62 | 0.95 |
| 43 | Recognize, understand and write common Chinese characters | 3.90 | 1.07 | 3.87 | 0.89 |
| 44 | Read for academic purpose | 3.78 | 1.11 | 3.80 | 0.89 |
| 45 | Write for academic purpose | 3.65 | 1.12 | 3.46 | 0.97 |
| 46 | Use information technology | 3.56 | 1.14 | 3.50 | 0.94 |
| 47 | Count and do arithmetic | 3.70 | 1.13 | 3.65 | 0.92 |
| 48 | Graphic geometry | 3.60 | 1.11 | 3.49 | 0.94 |
| 49 | Participate and conduct arts | 3.48 | 1.09 | 3.42 | 0.94 |
| 50 | Body or language imitation | 3.79 | 0.94 | 4.03 | 0.83 |
| 51 | Toy exploration | 3.75 | 0.95 | 3.95 | 0.84 |
| 52 | Play combination games | 3.78 | 0.92 | 3.89 | 0.82 |
| 53 | Play cause and effect games | 3.65 | 0.99 | 3.81 | 0.87 |
| 54 | Play functional games | 3.75 | 0.96 | 3.90 | 0.84 |
| 55 | Play symbolic games | 3.54 | 1.05 | 3.71 | 0.89 |
| 56 | Play parallel games | 3.64 | 0.99 | 3.79 | 0.87 |
| 57 | Play joint games | 3.62 | 1.01 | 3.79 | 0.88 |
| 58 | Play cooperative games | 3.68 | 1.05 | 3.85 | 0.89 |
| 59 | Responding to social concerns | 3.87 | 1.01 | 4.05 | 0.89 |
| 60 | Maintain balance in relationship | 3.86 | 1.00 | 4.01 | 0.90 |
| 61 | Share joint attention with others | 3.77 | 1.05 | 3.90 | 0.93 |
| 62 | Maintain interactions with others | 3.81 | 1.02 | 3.96 | 0.90 |
| 63 | Understand and infer the thoughts, intentions and feelings of others | 3.74 | 1.08 | 3.83 | 0.93 |
| 64 | Get along with others in familiar or public settings | 3.87 | 0.96 | 3.99 | 0.86 |
| 65 | Build and maintain healthy relationships with others | 3.84 | 1.01 | 3.94 | 0.87 |
| 66 | Recognize, attend to, and listen to the sounds of the environment | 3.92 | 0.95 | 4.08 | 0.85 |
| 67 | Respond to others | 4.08 | 0.93 | 4.20 | 0.85 |
| 68 | Can access information | 3.90 | 1.03 | 4.03 | 0.88 |
| 69 | Express needs, options, events, ideas and comments | 3.90 | 1.02 | 4.03 | 0.88 |
| 70 | Initiate and advance conversations | 3.77 | 1.11 | 3.84 | 0.92 |
| 71 | Use supportive skills in interactive conversations | 3.82 | 1.04 | 3.94 | 0.91 |
| 72 | Participate in group activities and be willing to do things for the group | 3.84 | 1.01 | 3.93 | 0.89 |
| 73 | Understand and follow group rules | 3.94 | 0.99 | 4.07 | 0.88 |
| 74 | Participate in labor practice activities | 3.87 | 1.05 | 3.89 | 0.90 |
| 75 | Has a sense of social belonging | 3.87 | 1.07 | 3.85 | 0.92 |
| 76 | Have readiness skills to enter the workforce | 3.76 | 1.16 | 3.93 | 0.95 |

# Table 2 *Differences between high and low groups of 76 key competency items*

| Items | Teachers | | Parents | |
| --- | --- | --- | --- | --- |
|  | t | p | t | p |
| 1 | 17.72 | 0.000 | 21.31 | 0.000 |
| 2 | 20.30 | 0.000 | 25.66 | 0.000 |
| 3 | 20.84 | 0.000 | 26.96 | 0.000 |
| 4 | 24.21 | 0.000 | 35.92 | 0.000 |
| 5 | 21.91 | 0.000 | 22.47 | 0.000 |
| 6 | 22.45 | 0.000 | 25.05 | 0.000 |
| 7 | 24.66 | 0.000 | 28.58 | 0.000 |
| 8 | 28.14 | 0.000 | 37.72 | 0.000 |
| 9 | 26.29 | 0.000 | 31.30 | 0.000 |
| 10 | 32.91 | 0.000 | 40.75 | 0.000 |
| 11 | 34.27 | 0.000 | 42.51 | 0.000 |
| 12 | 37.32 | 0.000 | 47.43 | 0.000 |
| 13 | 34.66 | 0.000 | 45.67 | 0.000 |
| 14 | 34.86 | 0.000 | 52.36 | 0.000 |
| 15 | 28.56 | 0.000 | 39.57 | 0.000 |
| 16 | 30.99 | 0.000 | 46.64 | 0.000 |
| 17 | 29.59 | 0.000 | 42.32 | 0.000 |
| 18 | 33.21 | 0.000 | 53.45 | 0.000 |
| 19 | 33.23 | 0.000 | 47.48 | 0.000 |
| 20 | 33.25 | 0.000 | 51.53 | 0.000 |
| 21 | 32.38 | 0.000 | 47.19 | 0.000 |
| 22 | 30.00 | 0.000 | 32.22 | 0.000 |
| 23 | 35.21 | 0.000 | 43.94 | 0.000 |
| 24 | 38.71 | 0.000 | 47.01 | 0.000 |
| 25 | 39.05 | 0.000 | 56.07 | 0.000 |
| 26 | 36.73 | 0.000 | 51.56 | 0.000 |
| 27 | 44.26 | 0.000 | 57.59 | 0.000 |
| 28 | 40.01 | 0.000 | 58.03 | 0.000 |
| 29 | 40.89 | 0.000 | 54.01 | 0.000 |
| 30 | 41.93 | 0.000 | 54.52 | 0.000 |
| 31 | 44.81 | 0.000 | 62.90 | 0.000 |
| 32 | 44.29 | 0.000 | 60.06 | 0.000 |
| 33 | 44.08 | 0.000 | 63.08 | 0.000 |
| 34 | 47.10 | 0.000 | 61.83 | 0.000 |
| 35 | 46.03 | 0.000 | 61.64 | 0.000 |
| 36 | 39.98 | 0.000 | 56.18 | 0.000 |
| 37 | 41.48 | 0.000 | 58.98 | 0.000 |
| 38 | 41.96 | 0.000 | 61.16 | 0.000 |
| 39 | 43.35 | 0.000 | 57.44 | 0.000 |
| 40 | 43.39 | 0.000 | 60.12 | 0.000 |
| 41 | 40.64 | 0.000 | 57.47 | 0.000 |
| 42 | 34.99 | 0.000 | 56.46 | 0.000 |
| 43 | 38.48 | 0.000 | 56.57 | 0.000 |
| 44 | 37.56 | 0.000 | 54.40 | 0.000 |
| 45 | 30.55 | 0.000 | 52.74 | 0.000 |
| 46 | 30.71 | 0.000 | 49.11 | 0.000 |
| 47 | 33.72 | 0.000 | 55.87 | 0.000 |
| 48 | 30.42 | 0.000 | 48.10 | 0.000 |
| 49 | 28.22 | 0.000 | 42.44 | 0.000 |
| 50 | 36.23 | 0.000 | 36.70 | 0.000 |
| 51 | 40.61 | 0.000 | 41.91 | 0.000 |
| 52 | 38.98 | 0.000 | 38.35 | 0.000 |
| 53 | 42.08 | 0.000 | 45.47 | 0.000 |
| 54 | 40.81 | 0.000 | 41.91 | 0.000 |
| 55 | 40.24 | 0.000 | 45.10 | 0.000 |
| 56 | 42.22 | 0.000 | 48.93 | 0.000 |
| 57 | 43.56 | 0.000 | 49.18 | 0.000 |
| 58 | 42.96 | 0.000 | 53.23 | 0.000 |
| 59 | 41.98 | 0.000 | 57.67 | 0.000 |
| 60 | 44.79 | 0.000 | 61.80 | 0.000 |
| 61 | 46.59 | 0.000 | 61.93 | 0.000 |
| 62 | 44.98 | 0.000 | 65.39 | 0.000 |
| 63 | 41.88 | 0.000 | 60.03 | 0.000 |
| 64 | 46.43 | 0.000 | 54.53 | 0.000 |
| 65 | 45.95 | 0.000 | 65.15 | 0.000 |
| 66 | 43.22 | 0.000 | 54.83 | 0.000 |
| 67 | 38.21 | 0.000 | 50.06 | 0.000 |
| 68 | 42.46 | 0.000 | 61.09 | 0.000 |
| 69 | 43.39 | 0.000 | 59.29 | 0.000 |
| 70 | 41.10 | 0.000 | 55.07 | 0.000 |
| 71 | 40.92 | 0.000 | 60.25 | 0.000 |
| 72 | 40.37 | 0.000 | 56.57 | 0.000 |
| 73 | 42.57 | 0.000 | 59.68 | 0.000 |
| 74 | 39.51 | 0.000 | 57.49 | 0.000 |
| 75 | 37.59 | 0.000 | 54.87 | 0.000 |
| 76 | 35.18 | 0.000 | 55.24 | 0.000 |

# Table 3 *Correlation between items and total score*

| Items | Teachers | Parents |
| --- | --- | --- |
| 1 | .407^***^ | .407*** |
| 2 | .478^***^ | .478^***^ |
| 3 | .499^***^ | .499^***^ |
| 4 | .604^***^ | .604^***^ |
| 5 | .422^***^ | .422^***^ |
| 6 | .452^***^ | .452^***^ |
| 7 | .507^***^ | .507^***^ |
| 8 | .625^***^ | .625^***^ |
| 9 | .578^***^ | .578^***^ |
| 10 | .675^***^ | .675^***^ |
| 11 | .667^***^ | .667^***^ |
| 12 | .718^***^ | .718^***^ |
| 13 | .699^***^ | .699^***^ |
| 14 | .747^***^ | .747^***^ |
| 15 | .668^***^ | .668^***^ |
| 16 | .717^***^ | .717^***^ |
| 17 | .677^***^ | .677^***^ |
| 18 | .780^***^ | .780^***^ |
| 19 | .747^***^ | .747^***^ |
| 20 | .773^***^ | .773^***^ |
| 21 | .747^***^ | .747^***^ |
| 22 | .565^***^ | .565^***^ |
| 23 | .693^***^ | .693^***^ |
| 24 | .706^***^ | .706^***^ |
| 25 | .800^***^ | .800^***^ |
| 26 | .788^***^ | .788^***^ |
| 27 | .789^***^ | .789^***^ |
| 28 | .798^***^ | .798^***^ |
| 29 | .783^***^ | .783^***^ |
| 30 | .791^***^ | .791^***^ |
| 31 | .833^***^ | .833^***^ |
| 32 | .830^***^ | .830^***^ |
| 33 | .833^***^ | .833^***^ |
| 34 | .795^***^ | .795^***^ |
| 35 | .811^***^ | .811^***^ |
| 36 | .772^***^ | .772^***^ |
| 37 | .805^***^ | .805^***^ |
| 38 | .803^***^ | .803^***^ |
| 39 | .791^***^ | .791^***^ |
| 40 | .828^***^ | .828^***^ |
| 41 | .816^***^ | .816^***^ |
| 42 | .815^***^ | .815^***^ |
| 43 | .796^***^ | .796^***^ |
| 44 | .783^***^ | .783^***^ |
| 45 | .782^***^ | .782^***^ |
| 46 | .770^***^ | .770^***^ |
| 47 | .796^***^ | .796^***^ |
| 48 | .753^***^ | .753^***^ |
| 49 | .721^***^ | .721^***^ |
| 50 | .636^***^ | .636^***^ |
| 51 | .675^***^ | .675^***^ |
| 52 | .642^***^ | .642^***^ |
| 53 | .716^***^ | .716^***^ |
| 54 | .663^***^ | .663^***^ |
| 55 | .735^***^ | .735^***^ |
| 56 | .754^***^ | .754^***^ |
| 57 | .757^***^ | .757^***^ |
| 58 | .784^***^ | .784^***^ |
| 59 | .810^***^ | .810^***^ |
| 60 | .816^***^ | .816^***^ |
| 61 | .833^***^ | .833^***^ |
| 62 | .833^***^ | .833^***^ |
| 63 | .824^***^ | .824^***^ |
| 64 | .758^***^ | .758^***^ |
| 65 | .825^***^ | .825^***^ |
| 66 | .758^***^ | .758^***^ |
| 67 | .722^***^ | .722^***^ |
| 68 | .823^***^ | .823^***^ |
| 69 | .820^***^ | .820^***^ |
| 70 | .813^***^ | .813^***^ |
| 71 | .808^***^ | .808^***^ |
| 72 | .805^***^ | .805^***^ |
| 73 | .813^***^ | .813^***^ |
| 74 | .814^***^ | .814^***^ |
| 75 | .792^***^ | .792^***^ |
| 76 | .800^***^ | .800^***^ |
| ^***^, P<0.001 | | |
